# Synaptic Efficacy as a Function of Ionotropic Receptor Distribution: A Computational Study

**DOI:** 10.1371/journal.pone.0140333

**Published:** 2015-10-19

**Authors:** Sushmita L. Allam, Jean-Marie C. Bouteiller, Eric Y. Hu, Nicolas Ambert, Renaud Greget, Serge Bischoff, Michel Baudry, Theodore W. Berger

**Affiliations:** 1 Center for Neural Engineering, Department of Biomedical Engineering, University of Southern California, Los Angeles, CA, United States of America; 2 Rhenovia Pharma, Mulhouse, France; 3 Graduate College of Biomedical Sciences, Western University of Health Sciences, Pomona, CA, United States of America; SUNY Downstate MC, UNITED STATES

## Abstract

Glutamatergic synapses are the most prevalent functional elements of information processing in the brain. Changes in pre-synaptic activity and in the function of various post-synaptic elements contribute to generate a large variety of synaptic responses. Previous studies have explored postsynaptic factors responsible for regulating synaptic strength variations, but have given far less importance to synaptic geometry, and more specifically to the subcellular distribution of ionotropic receptors. We analyzed the functional effects resulting from changing the subsynaptic localization of ionotropic receptors by using a hippocampal synaptic computational framework. The present study was performed using the EONS (Elementary Objects of the Nervous System) synaptic modeling platform, which was specifically developed to explore the roles of subsynaptic elements as well as their interactions, and that of synaptic geometry. More specifically, we determined the effects of changing the localization of ionotropic receptors relative to the presynaptic glutamate release site, on synaptic efficacy and its variations following single pulse and paired-pulse stimulation protocols. The results indicate that changes in synaptic geometry do have consequences on synaptic efficacy and its dynamics.

## Introduction

In the mammalian central nervous system synapses often differ significantly in terms of their potency, and synaptic strength at individual synapses can change significantly as a function of time and/or use. Most often, these sources of variance in synaptic strength have been attributed to presynaptic mechanisms [1], although postsynaptic mechanisms have been preferentially postulated to account for long-term changes in synaptic efficacy. Studies emphasizing postsynaptic factors, however, have given far less importance to the geometry of synapses, and in particular to the postsynaptic distribution of receptors and/or receptor sub-types. AMPARs, NMDARs and mGluRs are differentially located within the postsynaptic membrane [2], and exhibit different kinetics. Given that glutamate released from presynaptic vesicles diffuses across the synaptic cleft, it is reasonable to assume that postsynaptic receptors located at varying distances from the release site will see varying concentrations of glutamate. We therefore explored the functional consequences resulting from various subsynaptic localization of ionotropic glutamate receptors. As glial cells ensheath central synapses, their powerful glutamate uptake systems contribute to further regulate glutamate concentration within the synaptic environment [3]. Finally, various extracellular ions and exogenous compounds, such as magnesium and glycine, also regulate activation of NMDA receptors at these synapses. All these factors may influence the shape of synaptic responses. However, it is difficult to experimentally test the influence of such contributing factors on synaptic potency due to the difficulty in locally controlling multiple factors at single synapses. In this study, we use the EONS (Elementary Objects of the Nervous System) synaptic modeling platform, which was specifically developed to analyze the roles of synaptic geometry, and in particular the ionotropic receptors localization relative to the glutamate release site, and receptor kinetics, in the regulation of synaptic efficacy. This biophysical modeling approach allowed us to systematically vary the parameters, which is very hard to realize through conventional experimental techniques. In this study, we investigated the interactions between subsynaptic receptor location and receptor dynamics on glutamatergic synaptic responses. It was also of interest to evaluate the contribution of the nonlinearities that arise in response to multiple stimuli due to receptor desensitization. Desensitization is defined as the receptor state in which the receptor is not functional even when an agonist of the receptor is bound.

Understanding the influence of the spatial location of receptors is essential because clustering of glutamate receptors at synapses plays an important role during brain development and in synaptic plasticity of excitatory transmission. The surface trafficking of AMPA receptors either through insertion into the membrane of the postsynaptic density (PSD) or through lateral diffusion can account for tuning synaptic transmission [4], and receptor alignment with the presynaptic release site can account for mechanisms such as short-term potentiation (STP) and long-term potentiation (LTP) [5]. Similarly, synaptic morphology has been reported to undergo changes during LTP [6], which leads to alterations in receptor content and expression in dendritic spines. This phenomenon could also provide clues to understand how network stability might arise from modifications at the synaptic level and how mechanisms such as receptor localization may play a crucial role in affecting synaptic dynamics and efficacy.

Beyond understanding the contribution of receptor location to synaptic potency, it is also crucial to underscore that disruption of receptor location resulting from PSD (Post -synaptic Density) protein mutations and deletions may lead to functional disturbances, which have clear implications on the pathogenesis of autism spectrum disorders and epilepsy [7].

## Results

We calibrated the parameters of our modeling platform to replicate the reported behavior of CA1 hippocampal pyramidal neurons, which play a crucial role in propagating information to other layers of the hippocampus. Our results section begins with a systematic determination of synaptic response as a function of AMPAR location relative to the source of glutamate release for a single quantum elicited by the stimulation with a presynaptic (axonal) input pulse. In this section, our simulations determine the probabilities of the receptor to be in a desensitized or in open conducting state as a function of glutamate occupancy, and as a function of receptor location with respect to release site. Next, we apply the same input stimulation protocol to evaluate synaptic currents elicited by NMDAR activation and evaluate how their localization in the membrane influences their functional states. In addition, since AMPARs and NMDARs co-exist at most CA1 hippocampal synapses, we provide a few examples to demonstrate how EPSC scales as a function of the ratio of AMPAR to NMDAR, including Mg^2+^ concentration as a tuning factor to control scaling of EPSC responses. Relative timing between two input events is critical to determine if a receptor exhibits non-linear dynamics, i.e. generates a higher or lower response to the second event compared to the first. To study this non-linear range, we simulated ionotropic receptor responses to paired pulse input stimulus over a large range of interpulse intervals, from 10 msec to 2 s.

### A. Synaptic potency as a function of AMPAR location

We first focused on studying the influence of AMPAR location on synaptic potency. AMPARs were placed in concentric circular strips separated by 20 nm and distributed along the PSD from 0 to 200 nm away from the release site. AMPARs have 4 glutamate binding sites and require at least 2 glutamate molecules to be bound for the associated Na+ channel to open. Based on the number of glutamate molecules bound, the receptor transitions between different states, such as desensitized, deactivated, open, or closed. Many mathematical models describe the kinetics of AMPA receptor responses to glutamate binding [8, 9]. The AMPA receptor model used here [9], is an elaborate model built to capture desensitization effects induced by glutamate occupancy. This model incorporates the AMPAR desensitization states when 0,1,2,3 and 4 glutamate molecules are bound.

**Fig 1:**
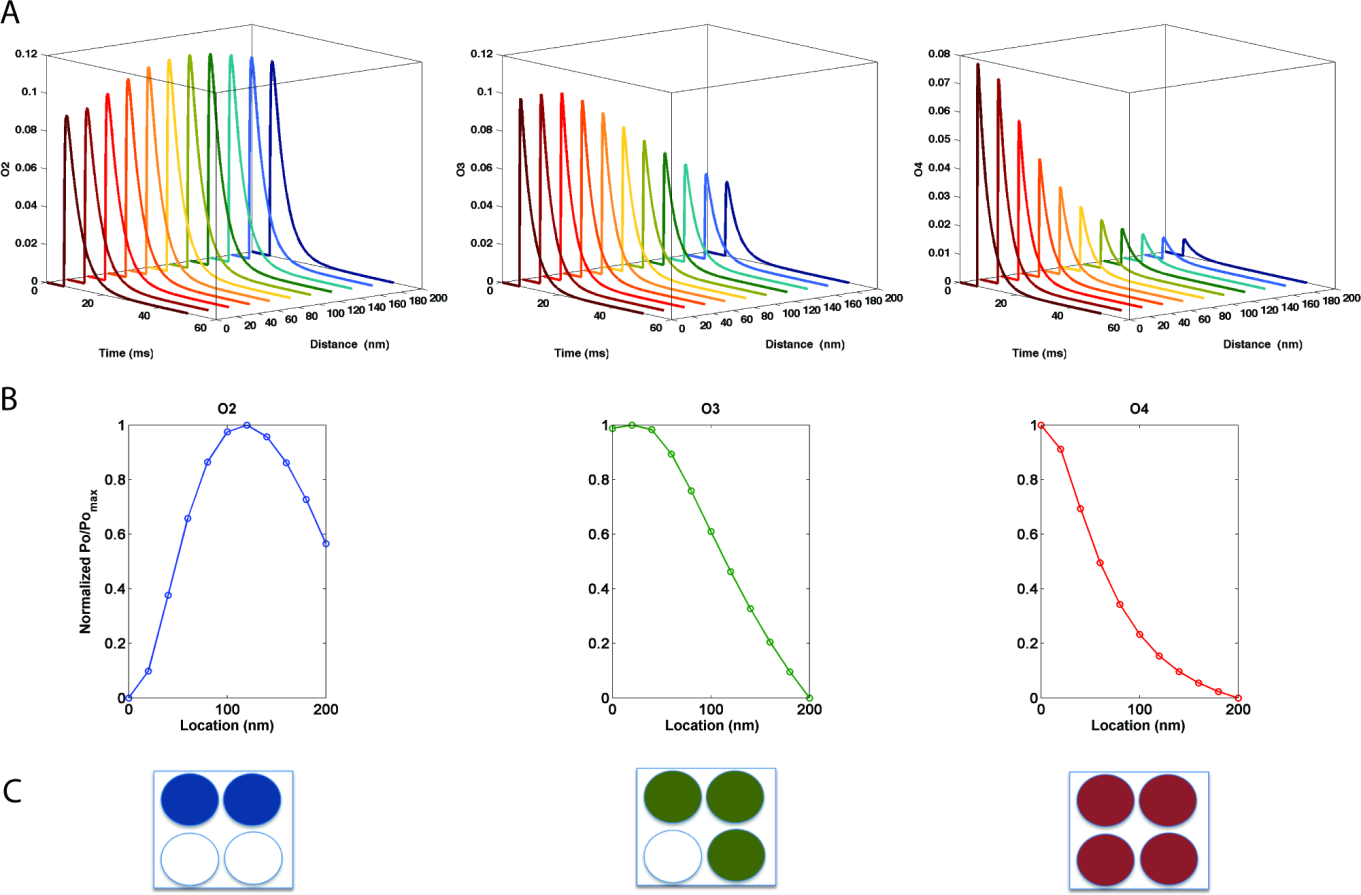
Probability of AMPAR open states as a function of receptor location. (Top row) Probability of AMPAR open states as a function of the distance from release site when 2, 3 and 4 glutamate molecules are bound (shown by filled circles—bottom row). Middle row shows the normalized maximum values scaled between 0 and 1 to highlight the location where the probability of open state is maximum.

In general, AMPAR localization has been reported to vary across the PSD [10, 11]. While the receptor could be occupied by ‘n’ molecules at any point in time (n = 0,1,2,3,4), the probability of the receptor being in various states may be described as a function of the receptor location relative to the source of glutamate release. AMPARs were activated by a single vesicular release event (elicited by a single presynaptic depolarization), and the location of the receptor was varied from 0 nm to 200 nm away from the release source. The results for O2, O3, and O4 states (open conducting states when two, three and four glutamate molecules are bound, respectively) are presented in Fig 1A (Top row).

As expected, the probability for the open states O2, O3, O4 varied as a function of location. For the O2 state, the maximum probability was reached when the receptor was located at 140 nm. For the O3 state, i.e. when 3 glutamate molecules are bound, receptors located around 20–40 nm exhibited a higher probability, while for the O4 state, receptors located closest to the glutamate source exhibited the highest probability.

Thus, our results indicated that each conducting state had a preferred location for which its probability was maximum. To show this effect more clearly, the ratios Po_n_/max(Po_n_) (n = 2,3,4 glutamate molecules bound) were plotted as a function of receptor location (Fig 1B). Fig 1C illustrates the number of glutamate molecules bound to the four available binding sites. The normalized maximum value is observed to shift towards smaller distances from the release site as the number of bound glutamate molecules increases. In addition to observed variations in individual state probability, the conductance associated with each open state (based on the number of glutamate molecules bound) is different. For this particular AMPAR model, the conductance associated with the O2 state is 9 pS, 15 pS for the O3 state and 21 pS for the O4 state. Consequently, the weighted sum of all these states generates currents that are dominated by the state O4, due to its highly weighted conductance of 21 pS.

**Fig 2:**
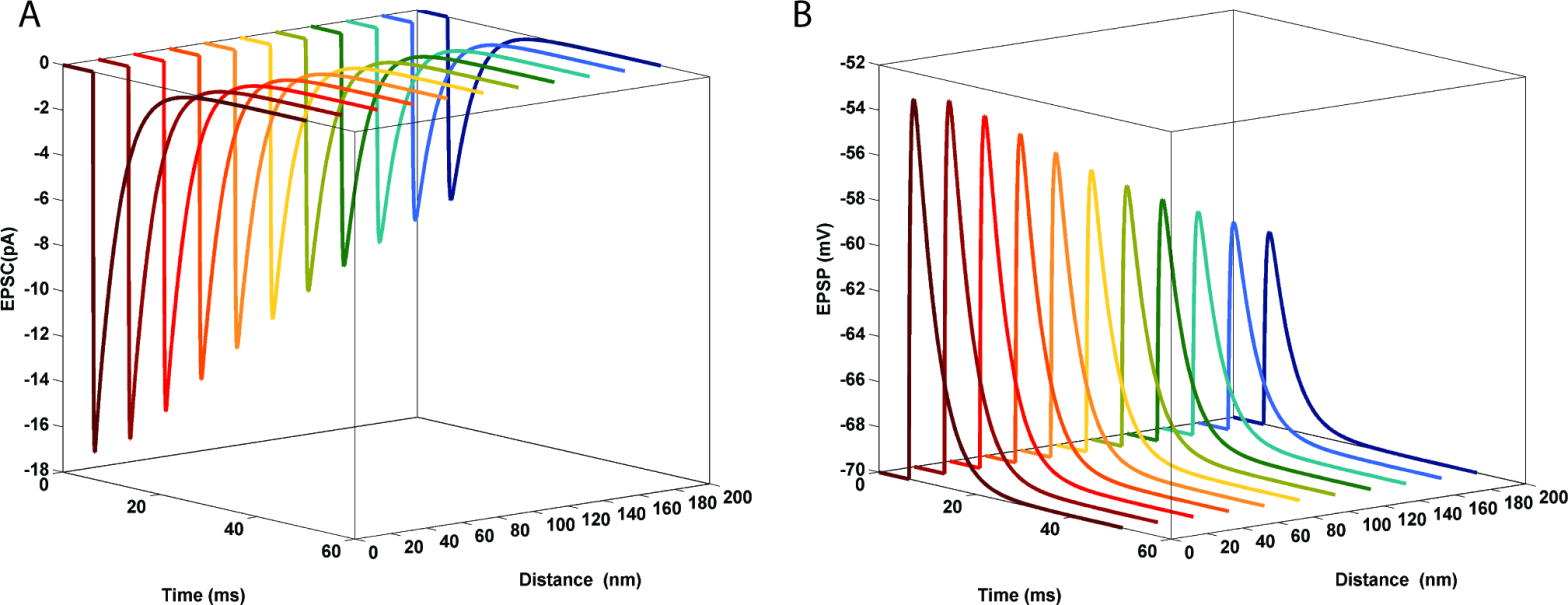
AMPAR-mediated EPSC as a function of the distance from the release site. AMPAR-mediated EPSCs to a single pulse. A gradual decrease in EPSC amplitude was observed when the distance between the release site and the receptors increases. The peak value of EPSC was obtained at 0 nm and was 52% larger than the peak amplitude when AMPARs are located 200 nm away from the release site (A). The resulting EPSP values as a function of the distance are shown in B. As expected the EPSPs mediated by AMPARs closest to the glutamate release site had the highest amplitude, 50% larger than the peak amplitude of AMPAR located 200 nm away.

Multiplying the weighted sum of conductance by the probability of each conducting state and the postsynaptic driving force generates AMPAR-mediated EPSC (see methodology section for the exact formula). Fig 2A and 2B illustrate resulting AMPAR-mediated EPSC and EPSP as a function of the distance between receptor and the release site. As expected, EPSC and EPSP responses elicited by AMPARs closest to the release site resulted in the maximum amplitude.

**Fig 3:**
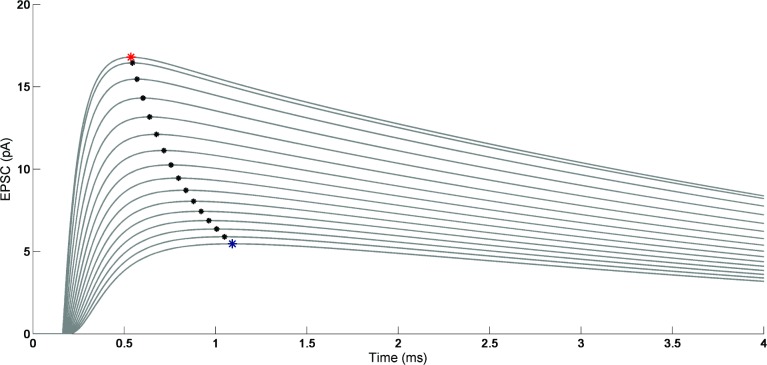
Time-to-peak of AMPAR-mediated EPSC as a function of the distance from the release site. Simulated EPSCs as a function of AMPAR location. The time-to-peak of EPSCs show a 0.5 ms delay when AMPA receptors are 300 nm (marked by blue asterisk) away, as compared to receptors located at 0 nm (marked by red asterisk).

To assess variations in corresponding time-to-peak responses, all EPSCs were plotted on the same time axis, as shown by the red markers (Fig 3). AMPA receptors located 300 nm away from the glutamate source exhibited a 0.5 msec delay for reaching the maximum amplitude of response compared to receptors located at 0 nm.

#### AMPA Receptor occupancy and Desensitization

**Fig 4:**
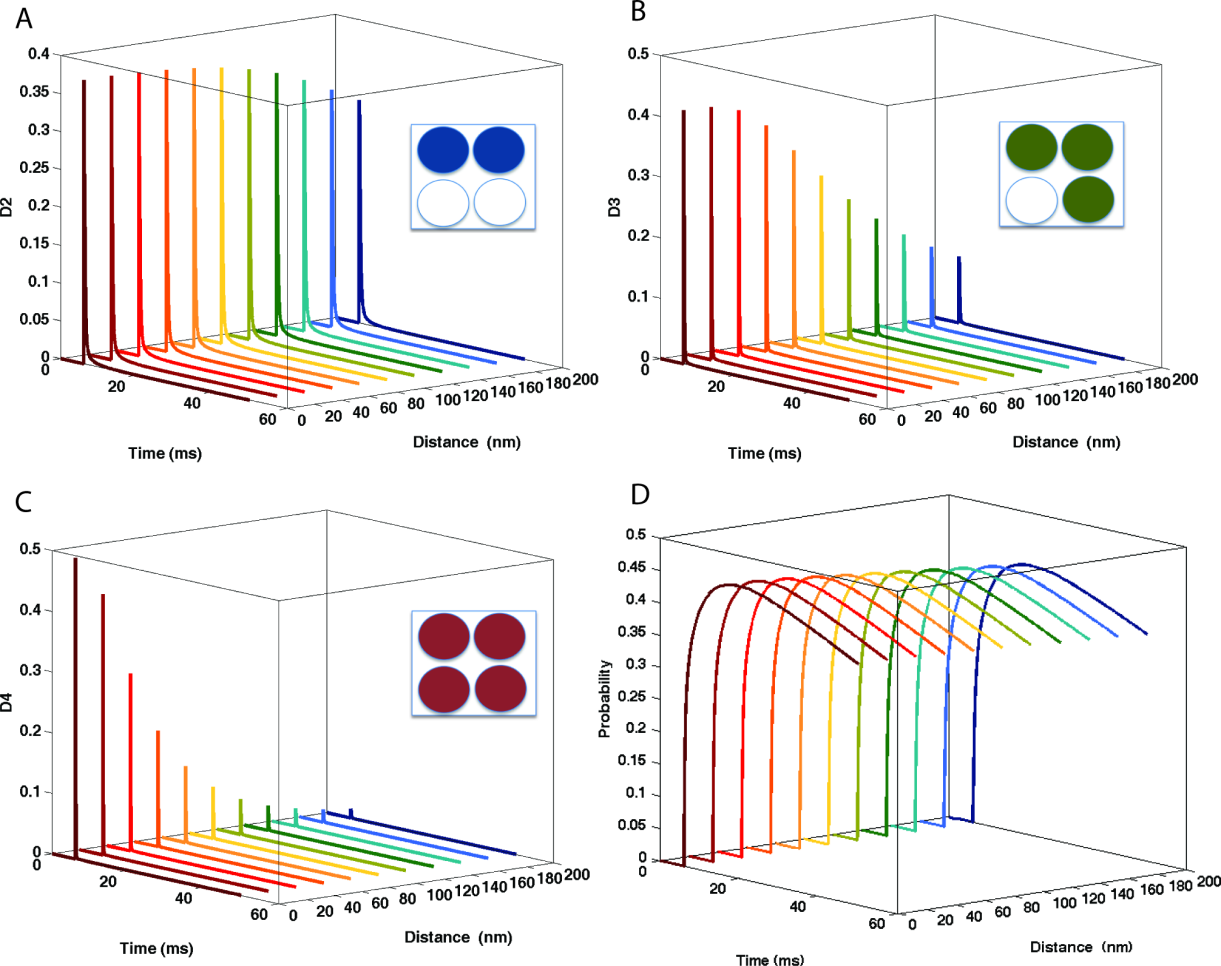
Desensitization of AMPAR as a function of the distance from the release site. Individual plots (from top) represent the probability of desensitization when (A) 2 glutamate molecules are bound, (B) 3 glutamate molecules are bound and (C) 4 glutamate molecules are bound. (D) Overall desensitization, as a sum of all individual desensitized states including when 1 glutamate molecule is bound and no molecules are bound. Overall desensitization (sum of all desensitized D states) is relatively similar across all locations, as the receptor is likely to be in the desensitization state D0 (when no glutamate are bound) for a longer period of time.

Receptor desensitization properties play an important role in the receptor’s dynamical response as they determine the time during which receptors cannot respond (or respond significantly less) to subsequent glutamate pulses. The individual states D2, D3, D4 (desensitized when two, three and four glutamate molecules are bound—Refer to S1 Fig for AMPAR probabilistic model) exhibited a large variability in desensitization responses (Fig 4). In particular, AMPARs closest to the release site had a high probability of being in D3 and D4 states. However, the sum of all the individual desensitized states, including the state when no glutamate is bound) did not vary significantly as a function of distance from release site (Fig 4D).

**Fig 5:**
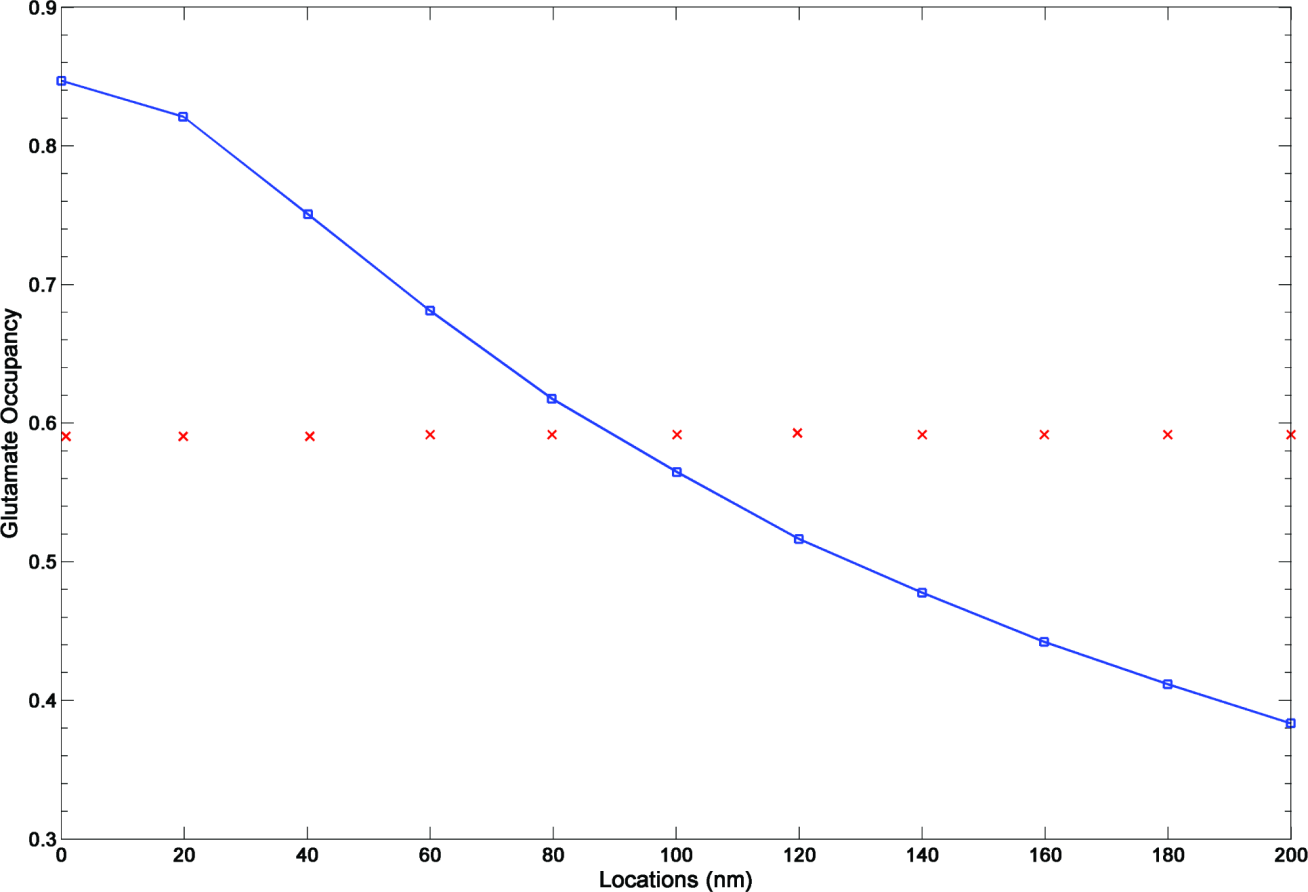
Probability of glutamate occupancy (peak values) as a function of the distance from the release site. The average AMPAR glutamate occupancy is 0.59.

Receptor occupancy is a key determinant of synaptic potency, and receptor saturation would limit the maximum synaptic responses at a given synapse. We thus calculated receptor occupancy based on binding probabilities of 0.5 when 2 out of 4 glutamate binding sites are occupied, 0.75 when 3 out of 4 binding sites are occupied and 1 when 4 out 4 binding sites are occupied, and weighted the sum of all states to obtain the values shown in Fig 5. The mean value of AMPA receptor occupancy for a single release event (indicated by red dots) across all locations was 0.59, consistent with the reported experimental values of peak open probability of 0.6 [8, 12].

### B. Synaptic potency as a function of NMDAR location

A similar analysis was conducted for NMDAR localization. NMDAR location was varied from 0 nm to 200 nm away from the source of release in steps of 20 nm. NMDAR-mediated EPSCs and distribution of receptor states, such as desensitization and open states, were determined. The NMDA receptor model we used was previously described (Ambert et al.); it has two binding sites for both glutamate and its co-agonist, glycine. It also incorporates receptor desensitization and has been validated using several experimental protocols [13, 14].

**Fig 6:**
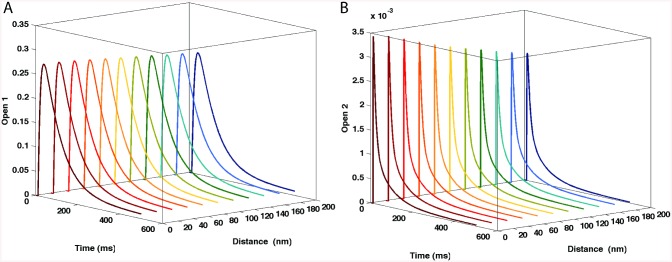
Probability of the two NMDAR open states as a function of the distance from the release site. There is a small variation in the individual open state probability as a function of distance. Open state 2 did show a slight variation as a function of receptor location, but the overall amplitude is very small (in the order of 10^−3^).

Probability of the two open states of NMDARs was analyzed as a function of NMDA receptor location. Unlike AMPARs, which demonstrated a location-dependent change in conductance, NMDARs did not exhibit significant variability in open conducting state probability (Fig 6A and 6B for open conducting states O1 and O2, respectively). The probability remained about 0.3 for NMDARs at different locations. Open state O1 has a conductance of 40 pS and O2 of 247 pS. The resulting conductance is the sum of the conductance values multiplied by their respective open state probabilities, also taking into account the voltage-dependent Mg^2+^ blockade of NMDAR channel and the driving force. The Mg^2+^ concentration used for these simulations was 1 mM.

**Fig 7:**
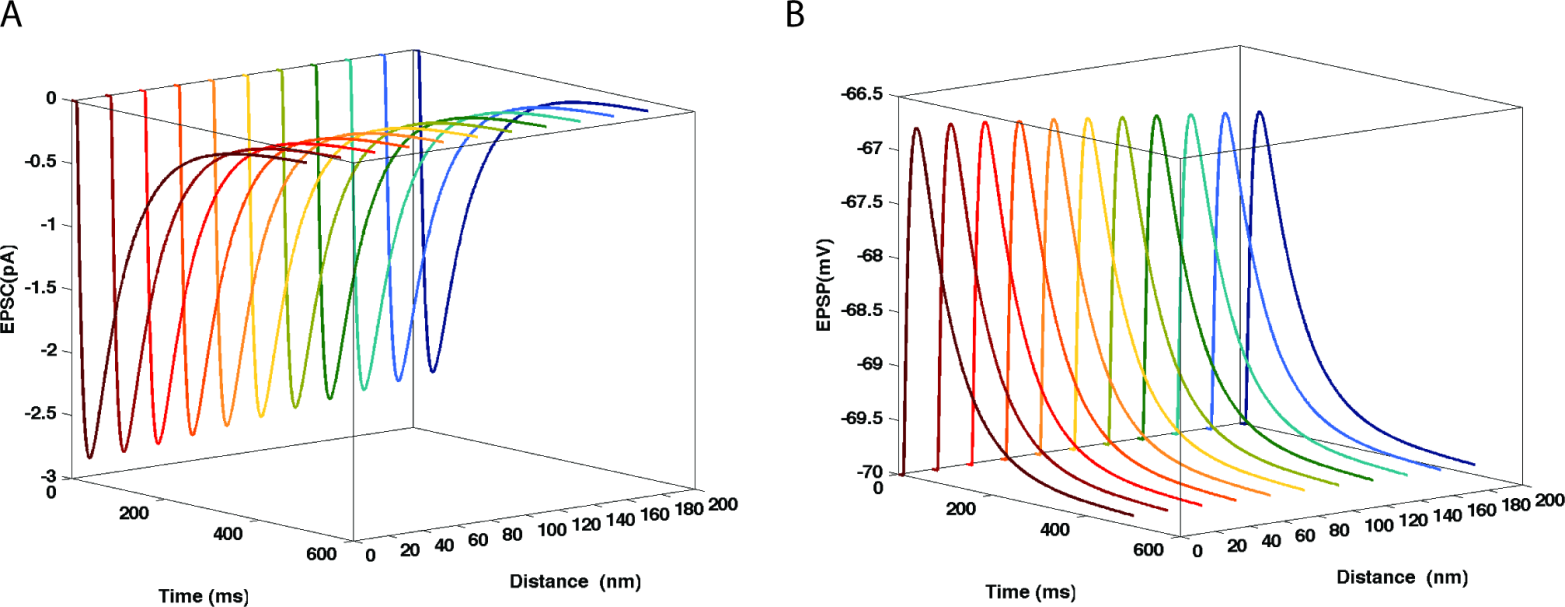
Lack of influence of NMDAR location on NMDAR-mediated EPSCs and EPSPs. NMDAR-mediated EPSCs (A) and EPSPs (B) as a function of NMDAR location.

Since experimental results indicate a range of 5–30 NMDA receptors per synapse [15], we used 20 NMDA receptors in our study to scale EPSC responses. NMDAR-mediated EPSCs as a function of receptor location are shown in Fig 7A. As previously reported [16, 17] simulation results indicated that NMDAR-mediated EPSP amplitudes were not location-dependent (Fig 7B); these results reflect the relatively high affinity of glutamate for NMDAR, which essentially eliminates the influence of location on receptor occupancy. We previously analyzed the effects of glial glutamate transporters on NMDAR kinetics [18], and showed that decay of NMDAR-mediated synaptic responses was inversely related to the density of glutamate transporters, although this effect was quantitatively small.

### C. Summation of AMPAR-mediated EPSCs and NMDAR-mediated EPSCs, influence of Mg^2+^ concentration

**Fig 8:**
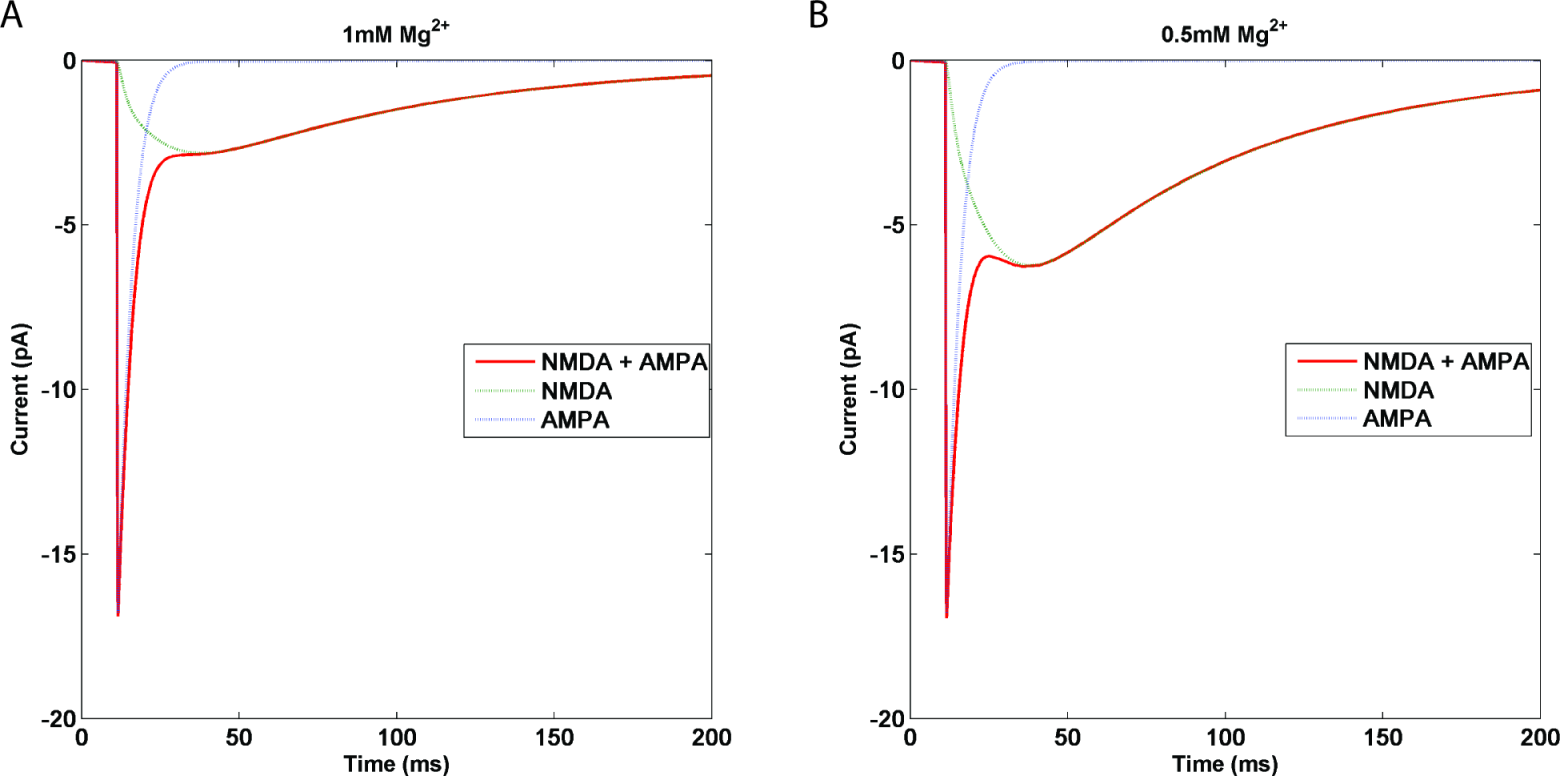
Summation of AMPAR- and NMDAR-mediated EPSCs at two magnesium concentrations. EPSCs (shown in red) are a summation of AMPAR (blue dotted line)-mediated and NMDAR-mediated currents (green dotted line). When Mg^2+^ concentration varies from 1 mM to 0.5 mM, NMDAR-mediated current is larger, changing the overall EPSC (in red).

In 85% of matured CA1 hippocampal synapses, AMPARs and NMDARs co-exist [19, 20]. The ratio AMPAR/NMDAR numbers and their respective conductance values are important determinants of EPSC waveform. EPSC quantal size and quantal content vary with the developmental stage of the animals; similarly, AMPAR expression increases and NMDAR subunit composition vary during postnatal development [21], resulting in EPSCs with a much sharper rise time in the adult. Other factors, such as extra-cellular magnesium concentration, shape NMDAR-mediated EPSC. As shown in Fig 8A, the fast-rising AMPAR-mediated component of the EPSC was comparatively larger when Mg^2+^ concentration was set to 1 mM, as Mg^2+^ blockade reduced NMDAR-mediated component of the EPSC. However, for Mg^2+^ concentration set at 0.5 mM, the EPSC had a predominant NMDAR-mediated component (Fig 8B). These results underscore the importance of variations in ionic extracellular concentrations on EPSC waveform. In the following section, we analyze the effects of Mg^2+^ concentration on EPSC waveforms elicited by paired-pulse stimulations.

### D. Paired-Pulse ratio as a function of AMPAR and NMDAR location

Neurons are highly non-linear spatio-temporal integrators, and the time interval between two action potentials may have a profound impact on postsynaptic responses. We analyze the effects of localization of both AMPARs and NMDARs on the overall synaptic response in response to a paired-pulse stimulation. From the synaptic response obtained, the paired pulse ratio is calculated as the ratio of the peak amplitude of the EPSC elicited by the second of two pulses divided by the peak amplitude of the first EPSC. Several presynaptic factors can change the response to the second input, although there is also evidence supporting a postsynaptic origin of paired pulse facilitation [22]. The increase or decrease in presynaptic release is attributed to many mechanisms, such as residual calcium, or vesicular depletion [23], while postsynaptic AMPAR properties could also regulate synaptic facilitation [24]. The facilitation or depression effect of the second postsynaptic response is also determined by the input time interval [25]. In this study, we used the same amount of neurotransmitter released for both pulses (See methods) in order to eliminate all presynaptic sources of variability. Therefore, changes in postsynaptic responses are solely the result of changes in postsynaptic receptor properties.

**Fig 9:**
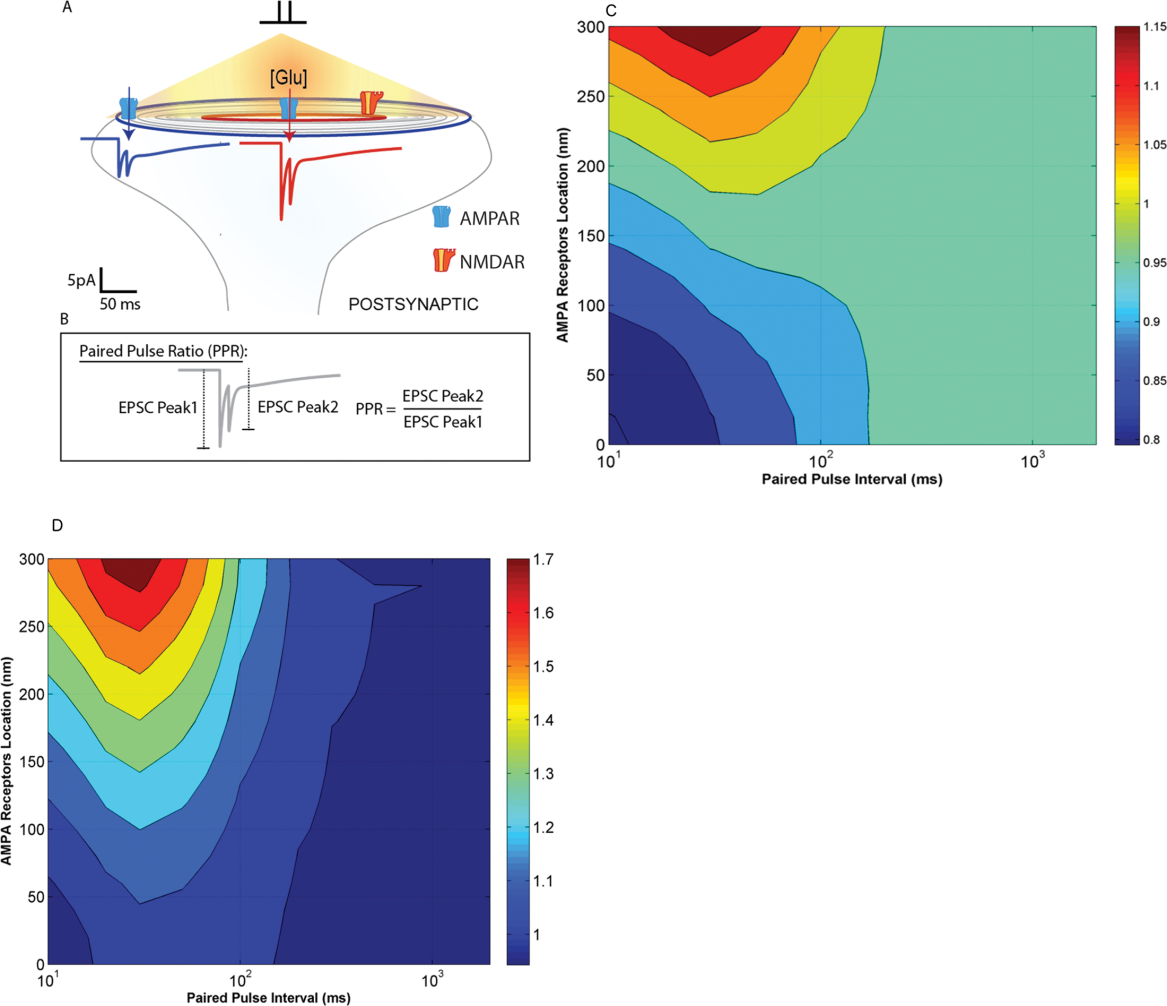
Schematic representation of the PSD and the receptors located on the postsynaptic membrane. **A**: Postsynaptic responses were elicited by a paired pulse stimulus with an inter pulse interval of 10 ms. The EPSC waveforms (shown in red and blue) are a result of ionic current flow through both AMPAR (located right opposite to the glutamate release site and at 200 nm. respectively) and NMDAR channels. The paired pulse ratio (PPR) as explained in Box B is the ratio of the maximum amplitude of the second response divided by the maximum amplitude of the first response. When AMPARs are closer to the release site, PPR is ~0.85 and when they are farther away, PPR is ~0.96. Paired pulse ratios (collected from 16x11 simulations—AMPARs located from 0 nm to 300 nm (16 data points along the x-axis) and for inter pulse intervals varying from 10 to 2000 ms (11 data points along the y-axis) simulations). **C: PPRs of synaptic EPSCs for [Mg** ^**2+**^ **] = 1mM**. Paired pulse ratios were plotted for AMPARs located from 0 nm to 300 nm (16 data points along the x-axis) and for inter pulse intervals varying from 10 to 2000 ms (11 data points along the y-axis) simulations). The paired pulse ratios vary between 0.8 and 1.18 (as indicated by the color bar). **D: PPRs of synaptic EPSCs for [Mg** ^**2+**^ **] = 0.5mM**. [Mg^2+^] was maintained at 0.5 mM to simulate the effect of partial unblocking of NMDA receptors by Mg^2+^. PPRs significantly varied with AMPAR locations between 1 to 1.7, more specifically for relatively small inter pulse intervals (10–100 ms).

We determined EPSC responses elicited by paired pulse stimuli with various inter-pulse intervals, as a function of receptor localization; EPSC responses elicited by paired pulse stimulation with 10 msec inter-pulse interval are shown in Fig 9A. When AMPARs are at 0 nm, the peak EPSC amplitude of the second pulse is 80% of the first pulse shown in red (Fig 9). However this difference in peak response between the first and second pulse decreases as AMPAR distance from the release site increased. When receptors are at 200 nm, the response to the second pulse was almost 96% of the first pulse response. This may be due to a gradual decrease in the amplitude of the first pulse response as a function of receptor location, since glutamate concentration decreases as distance increases.

Note that the EPSCs simulated here are summations of AMPAR- and NMDAR-mediated responses, and the peak of the second EPSC (generated by AMPAR activation) frequently superimposes to the EPSC generated by NMDAR activation due to the first stimulus.

In the following, AMPAR locations were varied from 0 to 300 nm in increments of 20 nm. NMDARs were held at a constant location since their location did not affect EPSC amplitudes (see section B above). AMPAR locations were varied along one axis. The simulations were repeated for inter-pulse intervals (IPI) of 10, 20, 30, 50, 100, 200, 300, 500, 1000 and 2000 msec. The paired-pulse ratio obtained for each stimulation was then plotted on the z-axis.

Paired-pulse ratios varied from 0.8, when AMPARs are located close to the release site, to 1.15 (Fig 9C), when AMPARs are located 300 nm away from the release site. Highest PPRs were predominant for paired-pulse interval of 30 msec and when receptors were located far from the release site. PPRs varied as a function of both AMPAR location and inter-pulse interval. We also used longer inter-pulse intervals (500, 1000 and 2000 msec), but we did not observe any location-dependent variation in paired-pulse ratios.

The same protocol was repeated at a reduced Mg^2+^ concentration of 0.5 mM. As expected, the paired pulse ratios were now distributed over a wider range, from 1.0 to 1.7 (Fig 9D).

## Discussion

In this study, we specifically focused on the effect of postsynaptic geometry, i.e. receptor localization with respect to release site, and how this affects synaptic response and temporal dynamics. We analyzed the effects of ionotropic receptor location on synaptic efficacy using the EONS synaptic modeling platform. In addition, we determined the effects of key parameters, such as Mg^2+^ on synaptic efficacy. Immunogold labeling studies have shown variations in subsynaptic localization of ionotropic glutamate receptors, with more centrally located NMDARs within the PSD and AMPARs more uniformly distributed across the PSD [15, 26]. Also, single particle tracking experiments have shown lateral movements of AMPARs [27]. This receptor movement in itself could be associated with synaptic maturation and tuning of synaptic transmission [28], indicating that receptor localization-dependent effects may influence synaptic function in different ways. Variability in postsynaptic currents could also be a result of such location-dependent effects of receptors.

(i) We first validated amplitude variations of AMPAR and NMDAR-mediated EPSCs as a function of their spatial locations in response to a single release event. Our results were qualitatively in good agreement with previously reported results [16], [7, 17, 29]. However, both simulation and experimental results obtained from freeze-fracture replica labeling studies in corticogeniculate and retinogeniculate synapses showed only minor influence of density and distribution of ionotropic receptors on postsynaptic responses [30]. Indeed, assuming a uniform distribution and density of AMPARs would eliminate variability of synaptic responses across individual synapses. In the present study, we varied AMPAR location systematically in increments of 20 nm along the postsynaptic membrane assuming imaginary concentric circles centered on glutamate release site. A single AMPAR response was multiplied by 80 (80 being the number of receptors) to match the quantal responses in the range 5–20 pA, as reported within single boutons [31]. Modeling response from one AMPAR and scaling it by a factor of 80 is equivalent to generating responses of 80 AMPARs, because the radial distance of activation is similar and they summate linearly. This modeling choice was made to avoid the computational burden of computing many ODEs.

(ii) The parametric structure of the modeling platform allowed us to explore the internal state receptor dynamics, including desensitization and receptor occupancy, as a function of receptor location. Our results indicate that AMPARs are sensitive to their position relative to the release site and exhibit an optimal distance for maximum open conducting state probability. Depending on the number of glutamate molecules bound to the receptor, glutamate concentration available to the receptor is a key determinant of glutamate receptor occupancy. This value varies as a function of glutamate uptake and glutamate spillover from neighboring synapses. For our simulation study, a single vesicular release architecture was used. This working hypothesis is based on evidence that most hippocampal synapses release one vesicle of glutamate release [32–34]. Moreover, though many studies point to multi-vesicular release [35–37], vesicles may not be released at the same time, and even if a presynaptic compartment had two vesicles in a readily-releasable state, what really determines receptor response is the glutamate concentration available at the receptor binding sites, which is determined by the location of the receptor, relative to the source of glutamate release.

(iii) AMPA receptor responses were more sensitive to spatial location, while NMDA receptors did not show much variation in their response as a function of their location, most likely due to their high binding affinity for glutamate and slower binding dissociation rate [14, 38]. Additionally only two glutamate binding sites are available on NMDARs, and variations in the number of glutamate molecules would only play a lesser role in NMDAR activation. On the contrary, we demonstrated that the AMPA receptor containing four glutamate binding sites generated a response that was dependent on occupancy and distance relative to release site (See Fig 1). The binding affinity of glutamate to receptors varies across receptor sub-types and their responses have been shown to be highly frequency-dependent [39]. Here we focused on a generic NMDAR present at hippocampal synapses. We did not delve into the details of receptor subtypes in this study, this will be the focus of future studies as the platform provides the flexibility to configure and use any receptor sub-type model.

(iv) The timing between successive inputs/release events is a critical determinant of receptor recovery and saturation. We used paired-pulse stimulation to determine synaptic efficacy by comparing the peak amplitude value elicited by the second pulse to the peak amplitude elicited by the first pulse. We tested the effects of location of AMPARs on synaptic facilitation or depression. We observed a marked facilitative effect (quantitatively measured by the PPRs of postsynaptic responses) for a time interval of 20–30 msec. This is likely due to the underlying dynamics of NMDAR-mediated responses, which have a rise time around 30 msec, (i.e. the maximum amplitude of the NMDAR-mediated current occurs around 30 msec after the beginning of the pulse). When AMPAR-mediated peak response elicited by the second pulse is added to the NMDAR-mediated amplitude during the rising phase, the second pulse response is indeed increased. When AMPAR-mediated peak occurs on the decay phase or the normal baseline, the second pulse response is decreased. When the time interval between the two pulses is very large, the receptor response is terminated and the peak amplitude elicited by both input pulses is similar, yielding a paired pulse ratio of 1. We observed a paired pulse ratio of 1 for all inter stimulus intervals ranging from 500 msec to 2000 msec, as the NMDAR response decay time for this model is 500 msec, in agreement with experimental data [14]. Another interesting property of the receptor that could explain variations in postsynaptic responses varying as a function of inter-pulse intervals is desensitization. In a previous study [18], we demonstrated that AMPARs enter into a desensitized state more rapidly for paired pulse stimuli with very small intervals, thus reducing its responsiveness to the second pulse of glutamate. The resulting reduced probability of open state decreases AMPAR-mediated responses. Similarly, NMDARs enter into a desensitized state and receptors require a longer time to return to their original (non-desensitized) resting state. This desensitization property reduces the probability of receptors to enter into open state(s) thereby decreasing their mediated responses to subsequent pulses for short time intervals, while they are able to return to their original resting state for longer time interval (>500 msec). The platform described here allows us to determine individual probabilistic states of receptors, thus providing in depth understanding of synaptic responses.

(v) In addition to the simple additive effects of EPSC responses from both ionotropic receptors, factors such as Mg^2+^ concentration have a direct influence on peak amplitude and time decay of the responses. This topic was explicitly discussed in other publications [18, 40]. These factors indirectly affect EPSC waveforms and facilitation and depression effects.

(vi) Changing the ratio of NMDA and AMPA receptors also increases response variability. During synapse formation, NMDARs outnumber AMPARs as evidenced by the changes in the number of silent synapses [41]. Under such conditions, location-dependent effects of receptor distribution will not contribute to postsynaptic variances because receptor location has almost negligible effect on NMDAR-mediated responses. For this simulation study we assumed a constant density of 80 AMPARs and 20 NMDARs per concentric circular ring, consistent with the reported numbers of NMDARs between 5 and 30 [11, 15]. This is usually a scenario encountered in adult synapses. Two-photon uncaging of glutamate is a technique that is used to map functional glutamate receptors at the level of individual synapses; such studies have shown that mushroom spines have larger and more complex PSDs and contain up to 40–140 AMPA receptors per spine; thin spines on the other hand are reported to contain a more sparse distribution [42]. Based on experimental observations, we used average values of receptors numbers.

(vii) Overall, it is the interplay between spatial location of AMPAR, density and conductance of these channels combined with the pre-synaptic pattern of activity that influence synaptic potency and dynamics.

We acknowledge that the glutamate transients are much more rapid in 3D models, and 3D diffusion models may therefore be more accurate, but the error between the glutamate concentration profiles simulated by 2D and 3D diffusion models within the first 0.5msec is less than 5%. There is no glutamate left in the cleft after 0.5msec. The synaptic modeling platform described here is a component of the larger framework of neuron-synapse integrated simulation environment within which the information exchange across several layers of neuronal hierarchy is mediated by molecular mechanisms, and synaptic inputs drive neuronal spiking. The modeling components used within the scope of this study are validated with experimental data. As outlined above, the 2D diffusion model used here constitutes a reasonable approximation of the 3D diffusion model. Indeed there are a very few attempts known that incorporate mechanisms at several layers of the neuronal hierarchy. The complexity of the model chosen is a trade-off between the level of necessary details and computation time of the simulations.

This study underscores the importance of ionotropic receptor location along the postsynaptic membrane and quantitatively illustrates how this location alters synaptic responses. Our results support the idea that alterations in synaptic structure and the underlying receptors distribution contribute to synaptic plasticity. Synapses act as interfaces for neuronal information processing. Alterations in subsynaptic architecture due to neurological disorders may cause disruptions to structural machinery, will eventually lead to alterations in neuron spike processing abilities. The computational synapse model developed here and its integration into a morphologically realistic neuron model within our integrated multi-scale framework [43] will enable us to achieve greater insight into how ionotropic receptors distribution can influence neuronal spike timing. This will be the focus of our future work. Identifying these subtleties in peak amplitudes and time-to-peak response of EPSCs and EPSPs, paired pulse facilitation and depression effects at synapses that arise due to minor variations in the distribution of receptors might eventually provide clues to the functional diversity of synapses that arise from changes in receptor expression and distribution.

## Methodology

### EONS Synapse Model

EONS (Elementary Objects of the Nervous System) is a synaptic parametric modeling platform available online at http://synapticmodeling.com [43]. The diffusion and AMPA-R models are accessible on ModelDB website (Accession number: 184410). A parametric model is defined by a finite dimensional parameter space. This platform was developed to help better understand how changes in parameters of the elements of glutamatergic synapses, such as receptor distribution and density, relative position of postsynaptic receptors with respect to the release site, and ionic concentrations within the synaptic environment, influence synaptic responses.

**Fig 10:**
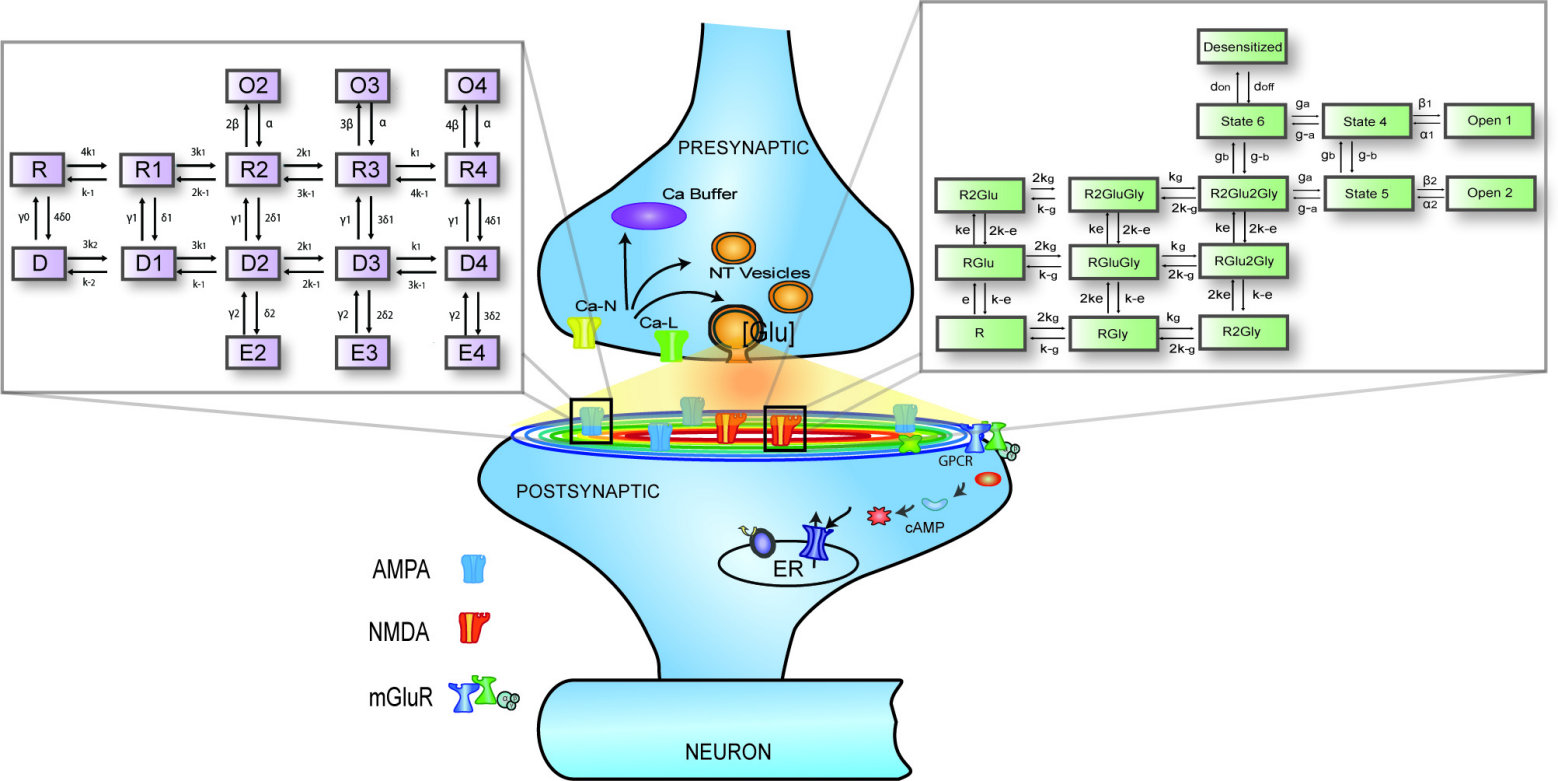
Schematic representation of the glutamatergic synapse model used in EONS. Some of the key elements are highlighted and details of the models of glutamate ionotropic receptors represented with kinetic rate constants. **Top Left**: Schematic of 16-states AMPA receptor model, adapted from Robert and Howe (2003). **Top Right**: Kinetic schema of the 15 states NMDA receptor model with kinetic rate constants, adapted from Ambert et al., 2010.

Fig 10 illustrates some of the mechanisms and elements that are incorporated in the current synaptic model: presynaptic calcium buffers, voltage-dependent calcium channels, a single vesicular release site for glutamate, glutamate diffusion in the synaptic cleft, Markov models of postsynaptic ionotropic and metabotropic receptors, which describe the dynamic behavior of receptors following glutamate binding. AMPARs are responsible for fast synaptic transmission, while NMDARs generate an EPSC with slower kinetics. In addition to the complexity at the synaptic level, NMDARs are highly nonlinear, as their activity is influenced by changes in magnesium and glycine concentrations and membrane potential. Other second messenger mechanisms are triggered by activation of metabotropic glutamate receptors (mGluR), which leads to a cascade of calcium-mediated events, which play a major role in short and long-term potentiation of synaptic transmission.

This simulation platform is implemented in JAVA, and a library of elementary models is available within the platform. Each elementary model was developed in SBML (Systems Biology Markup Language), using Cell Designer^TM^. Once tested and validated with several protocols, these elementary models were integrated into EONS, where they interact with each other, i.e., one model’s output may represent the input to another model.

For example, the elementary models of the receptors include various transition states of the receptors, such as inactive, desensitized and open states. The input to these models is the local glutamate concentration available at the receptor site. The time-dependent evolution of the receptor states is calculated by solving the system of ODEs describing the kinetics of the transition between states of the receptors. Only a few models relevant to this study will be presented in detail in this document. The features of the modeling platform are separated in four sections: (i) a presynaptic component (ii) glutamate diffusion in the synaptic cleft (iii) a postsynaptic component and (iv) a set of extrasynaptic components.

#### Presynaptic Component

Action potentials invade the presynaptic terminals and depolarize the membrane, activating voltage-dependent calcium channels (VDCCs). L, N and T calcium channels account for most of the Ca^2+^ influx in this component [44, 45]. Presynaptically, calcium buffers, mitochondria and various calcium pumps regulate the kinetics of calcium concentration following an action potential, which in turn trigger glutamate release [46, 47]. EONS provides the possibility of changing the location and number of presynaptic calcium channels away or towards the release site, which influence the amount of release [48]. Quantal release is highly stochastic in nature, though this feature is currently not modeled, and all simulations performed in this study used deterministic release, i.e., for the same presynaptic stimulus, the amount of glutamate released was always the same. This choice was made to avoid any variability due to presynaptic noise, since the focus of the present study was to determine the influence of the postsynaptic distribution of receptors on synaptic transmission.

#### Glutamate Diffusion

The diffusion model used here to calculate glutamate concentration inside the synaptic cleft as a function of the distance of the receptor from the release site was adapted from [47] using (Eq 1), and a diffusion coefficient of 0.4μm^2^ms^-1^. Though the theoretically calculated diffusion coefficients range from 0.2μm^2^ms^-1^ to 1μm^2^ms^-1^, we have chosen a value consistently found in literature to be one-third the value of glutamate diffusion in free solution [49]. The total number of transmitter molecules released by one vesicle was set at 3,000. The concentration of glutamate inside the cleft was determined by the following equation:

$$Glu\left(r,t,Q,D,\delta \right)=\frac{Q}{4\pi \varepsilon Dt}e^{\frac{-r^{2}}{4Dt}}$$ (1)

Where *Glu*, *r* and *D* represent the concentration of glutamate inside the synaptic cleft, the radial distance and the diffusion coefficient, respectively. *Q* represents the number of glutamate molecules released instantaneously, and ‘*δ*’ the height of the cleft, which was maintained constant throughout the simulations at 20 nm. The glutamate profile seen by postsynaptic receptors located at various distances from the release site is shown in Fig 10. We have demonstrated the scaling of EPSCs mediated by both AMPAR and NMDAR responses for various diffusion coefficients. In the main manuscript, all simulations were conducted with a diffusion coefficient = 0.4 μm^2^/ms^-1^. The S1 Fig illustrates the AMPAR and NMDAR mediated EPSCs obtained using diffusion coefficients 0.1 and 1μm^2^/ms^-1^. We observe that the changes in peak synaptic currents as a function of receptor location are more significant at lower diffusion coefficient = 0.1 um^2^ms^-1^.

#### Postsynaptic Components

At most CA1 hippocampal synapses, EPSCs are produced by the summation of ionic currents mediated by AMPARs and NMDARs. Each of these receptors has channel-forming subunits, and the differences in sequences and structures of these different subunit types result in different kinetic properties [50]. For AMPARs, we used the properties of GluR1 subunits, while we used NRA subunits for NMDARs, as they are the most abundant in CA1 synapses. However, other sub-types/receptor models with different kinetic parameters can be easily plugged into EONS. All the models kinetics were calibrated to experimental results obtained at room temperature.

#### AMPAR Model

We used the AMPA receptor model described in detail in [9], which represents a 16-states model describing transitions between resting, desensitized and open states (Fig 10). The rate constants for this model are listed in Table 1. Successive binding of 2, 3, and 4 glutamate molecules produces conformational changes leading to fast opening and closing of the channel.

**Table 1:** Kinetic parameters of AMPAR model used in EONS.

Parameter	Values
k1	10 mM^-1^ms^-1^
k-1	7 ms^-1^
k2	10 mM^-1^ms^-1^
k-2	4.1e^-4^ ms^-1^
γ_o_	0.001 ms^-1^
δ_o_	3.3e^-6^ ms^-1^
γ_1_	0.42 ms^-1^
δ_1_	0.017 ms^-1^
γ_2_	0.2 ms^-1^
δ_2_	0.035ms^-1^
Β	0.55 ms-^1^
Α	0.3 ms^-1^

The current through the channel is calculated by:

$$I_{AMPA}=nb_{AMPA}\left(g2\times O_{2}+g3\times O_{3}+g4\times O_{4}\right)\left(V-V_{rev}\right)$$ (2)

$${\dot{O}}_{i}=\left[M\left(Glu\right)\right].O_{i}$$ (3)

where *I*_*AMPA*_ is the current mediated by AMPARs, *nb*_*AMPA*_ is the number of AMPARs (in this study *nb*_*AMPA*_ is 80), consistent with reported AMPAR numbers between 46–147 at CA1 hippocampal synapses [42]. *g2*, *g3*, *g4* are unitary conductances associated with the channel in open states when 2, 3 and 4 glutamate molecules are bound with values of 9, 15 and 21 pS, respectively. The probabilities for the *O*_*2*,_ *O*_*3*,_ *O*_*4*_ states are calculated based on ODEs solved using SBML (Systems Biology Markup Language). Different ODE solvers are used in the EONS modeling platform. The derivatives of open states *O*_*i*_ (where *i* = 2, 3, 4) are calculated as a product of matrix M containing the other state transition rate constants with input *Glu* and vector of currents states *O*_*i*_. *Vrev* is the reversal potential of AMPAR (*Vrev* is 0 mV) and *V* is the membrane potential.

#### NMDAR Model

The NMDAR model consists of a 15-state kinetic scheme (Fig 10), which includes agonist (glutamate) and co-agonist (glycine) binding sites, channel blockers (memantine and magnesium), as well as several antagonist sites. The rate constants for this model are listed in Table 2. The kinetic parameters of this model are the same as in [40]. Results from various experimental protocols were used to validate this NMDAR model. For a single short pulse of glutamate, experimental results reported in [13] were used, whereas for long or repetitive glutamate inputs to the model, kinetic parameters were adjusted to properly capture effects of desensitization and to match experimental data from [51].

**Table 2:** NMDAR model parameters used in EONS.

Parameter	Values
k_e_	8.3 mM^-1^ms^-1^
k_-e_	0.0263 ms^-1^
k_g_	10 mM^-1^ms^-1^
k_-g_	0.0291 ms^-1^
g_b_	0.0671 ms^-1^
g_-b_	0.15 ms^-1^
g_a_	2.03 ms^-1^
g_-a_	22.8 ms^-1^
β_1_	35.2 ms^-1^
α_1_	0.728 ms^-1^
β_2_	0.787 ms-^1^
α_2_	11.2 ms^-1^
d_on_	0.03 ms^-1^
d_off_	9.5e^-4^ ms^-1^

The equations to calculate NMDAR-mediated synaptic current are:

INMDA=nbNMDA(Io1+(Mg2+Ko)e−δzFφmRT) (4)

$$I_{o}=g\left(V-V_{rev}\right)\left(O\left(t\right)\right)$$ (5)

$$g=g1+\frac{g2-g1}{1+e^{\alpha \varphi \text{m}}}$$ (6)

where *I*_*NMDA*_ is the current mediated by NMDARs. *nb*_*NMDA*_ was set at 20 for this study, consistent with experimental values [11, 15]. *I*_*o*_ is the current associated with the open conducting state *O(t)*, *which was* calculated using ODEs solved with kinetics described in [40]. The magnesium concentration in the external solution was set at 1 mM; *Ψm is* the electrical distance of the magnesium binding site from the outside of the membrane (set at 0.8); *R*, the molar gas constant (8.31434 J.mol^−1^.K^−1^); *F*, the Faraday constant (9.64867.104 C.mol^−1^); *T*, the absolute temperature (273.15°K); *g*_*1*_ and *g2 are* the conductances associated with the open states when one or two glutamate molecules are bound (40 pS and 247 pS respectively); and *α* = 0.01 is the steepness of the voltage-dependent transition from *g1* to *g2*.

The EPSCs thus obtained are a summation of responses of currents mediated by AMPARs and NMDARs.

## Supporting Information

S1 Fig AMPAR-mediated and NMDAR-mediated EPSCs as a function of the distance from the release site at diffusion coefficients = 0.1 μm^2^/ms^-1^ and 1 μm^2^/ms^-1^. (A)AMPAR-mediated EPSCs to a single pulse at a diffusion co-efficient of 0.1 um^2^ms^-1^. (B)AMPAR-mediated EPSCs to a single pulse at a diffusion co-efficient of 1 um^2^ms^-1^. (C)NMDAR-mediated EPSCs to a single pulse at a diffusion co-efficient of 0.1 um^2^ms^-1^. (D)NMDAR-mediated EPSCs to a single pulse at a diffusion co-efficient of 1 um^2^ms^-1^. For a higher diffusion coefficient AMPAR mediated EPSCs scaling as a function of receptor locations was noticeably more significant than with a diffusion coefficient = 0.1 um^2^ms^-1^. However NMDAR mediated EPSCs scaling as a function of receptor location was only observed at a higher diffusion coefficient 1 um^2^ms^-1^.
(PDF)
