# Supplementary figures and images for: Synaptic Efficacy as a Function of Ionotropic Receptor Distribution: A Computational Study
Source: PLoS One. 2015 Oct 19;10(10):e0140333. doi: 10.1371/journal.pone.0140333 (PMC4610697; doi:10.1371/journal.pone.0140333)

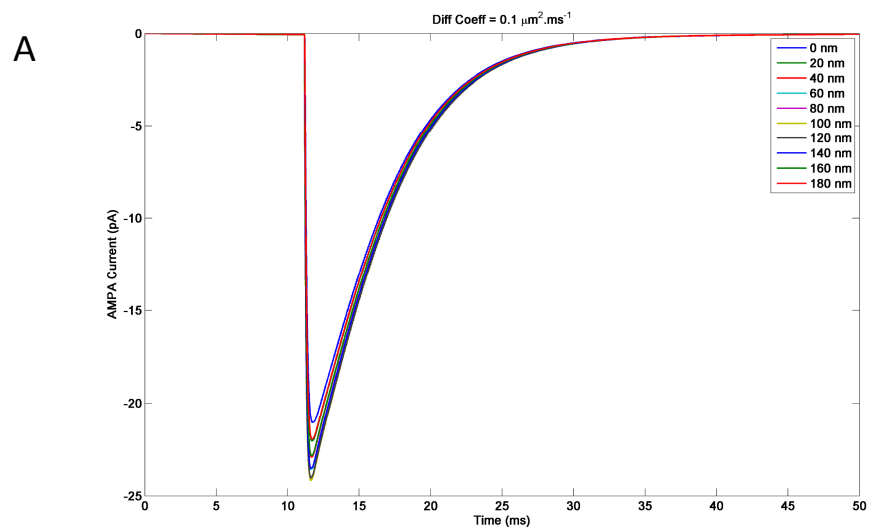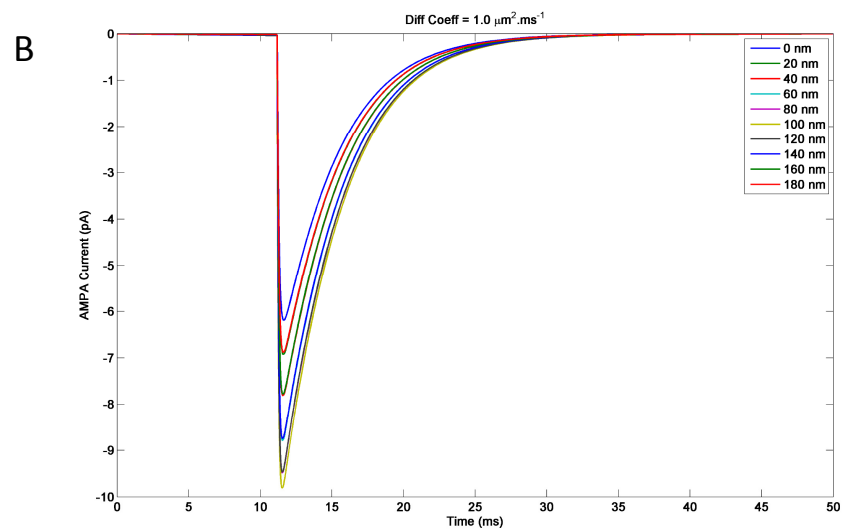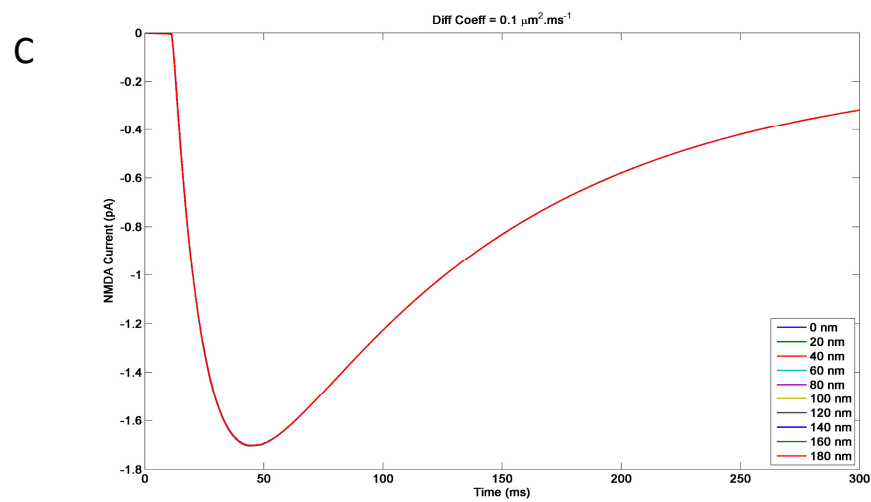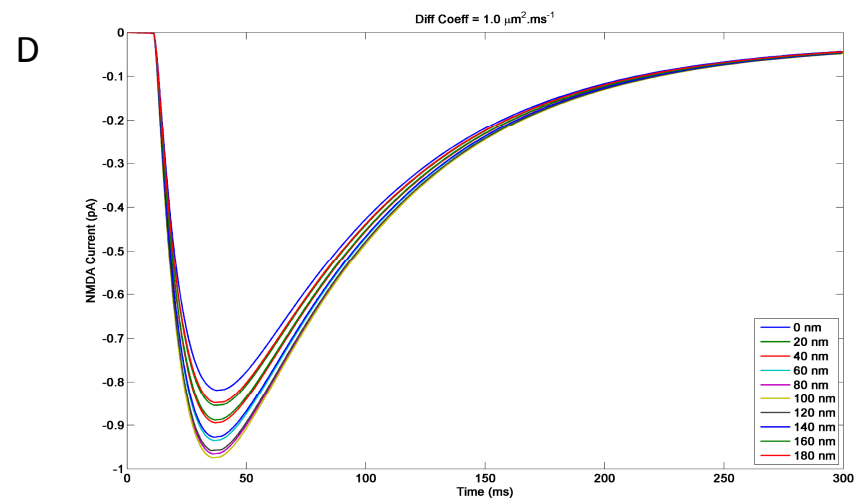

Supplement: S1 Fig — (A)AMPAR-mediated EPSCs to a single pulse at a diffusion co-efficient of 0.1 um2ms-1. (B)AMPAR-mediated EPSCs to a single pulse at a diffusion co-efficient of 1 um2ms-1. (C)NMDAR-mediated EPSCs to a single pulse at a diffusion co-efficient of 0.1 um2ms-1. (D)NMDAR-mediated EPSCs to a single pulse at a diffusion co-efficient of 1 um2ms-1. For a higher diffusion coefficient AMPAR mediated EPSCs scaling as a function of receptor locations was noticeably more significant than with a diffusion coefficient = 0.1 um2ms-1. However NMDAR mediated EPSCs scaling as a function of receptor location was only observed at a higher diffusion coefficient 1 um2ms-1. (PDF) [file pone.0140333.s001.pdf]
